# Shrub Encroachment: A Catalyst for Enhanced Soil Nutrients Storage in the Altai Mountains

**DOI:** 10.3390/plants14040623

**Published:** 2025-02-19

**Authors:** Xuexi Ma, Lianlian Fan, Abbas Fakher, Yaoming Li, Jiefei Mao, Meiniu Yang, Meng Yan, Bo Zhang, Yingzhi Gao

**Affiliations:** 1 Key Laboratory of Grassland Resources and Ecology of Western Arid Desert Area of the Ministry of Education, College of Grassland Science, Xinjiang Agricultural University, Urumqi 830052, China; maxx@ms.xjb.ac.cn (X.M.); ymeng324@163.com (M.Y.);; 2 Xinjiang Institute of Ecology and Geography, Chinese Academy of Sciences, Urumqi 830011, China; flianlian@ms.xjb.ac.cn (L.F.); sherazi@ms.xjb.ac.cn (A.F.); lym@ms.xjb.ac.cn (Y.L.); mjf@ms.xjb.ac.cn (J.M.); yangmeiniu23@mails.ucas.ac.cn (M.Y.); 3 Research Center for Ecology and Environment of Central Asia, Chinese Academy of Sciences, Urumqi 830011, China; 4 Key Laboratory of Vegetation Ecology of the Ministry of Education, State Environmental Protection Key Laboratory of Wetland Ecology and Vegetation Restoration, Institute of Grassland Science, Northeast Normal University, Changchun 130024, China

**Keywords:** Altai Mountains, ecological restoration, ecosystem processes, shrub encroachment, grassland types

## Abstract

Shrub encroachment in grasslands has a major impact on soil carbon storage (SOC_S_) and soil total nitrogen (STN_S_), which affects nutrient cycling and ecosystem processes. We explored the effects of shrub encroachment on SOC_S_ and STN_S_ in five grassland types in the Altai Mountains: mountain meadows, temperate meadow steppe, temperate steppe, temperate steppe desert, and temperate desert steppe. Shrub encroachment considerably improved SOC_S_ and STN_S_, with the greatest increases occurring in locations with high encroachment. The interaction between grassland type and encroachment extent also significantly influenced soil properties, including bulk density, soil water content, and microbial carbon and nitrogen. Specifically, SOC_S_ increased by 16%, 77%, and 129%, and STN_S_ increased by 43%, 94%, and 127% under low, medium, and high shrub encroachment, respectively. The soil stoichiometry shifted, with C/N ratios decreasing and C/P and N/P ratios increasing with shrub encroachment. Structural equation modeling (SEM) revealed that shrub encroachment indirectly affected SOC_S_ and STN_S_ through changes in soil nutrients and climate. Our findings suggest that shrub encroachment promotes soil C sequestration and alters soil nutrient cycling, with implications for grassland management and ecological restoration in the face of global climate change.

## 1. Introduction

The growth of native shrub cover, density, or biomass in grass ecosystems, frequently coupled with a decrease in herbaceous plant cover and density, is referred to as grassland shrub encroachment [1,2,3]. This process has greatly impacted grassland ecosystems over the last 150 years, increasing their multifunctionality. Ecosystem services, such as productivity and nutrient cycling, are impacted by shrub encroachment, which also affects important ecological processes [4,5,6]. The growth of the grassland economy and the safety and environmental stability of grassland ecosystems are at risk due to the changes in vegetation cover types and land use patterns brought about through the intensification of shrub encroachment. Shrub encroachment is now a major ecological concern on a worldwide scale.

Furthermore, shrub encroachment can act as a terrestrial carbon sink, altering regional and global carbon and nitrogen cycles [7,8,9]. The deep roots and litter accumulation of shrubs influence soil material intake and decomposition by altering plant composition, community structure, and microbial activity, eventually influencing soil carbon and nitrogen storage [8,10]. Current research shows that shrub encroachment has positive, negative, and neutral effects on soil carbon and nitrogen storage [1,10,11,12]. These disparities could be attributed to variances in shrub species, shrub encroachment construction methods, research procedures, and climatic region [13,14]. The relationship between soil heterogeneity and vegetation distribution is complex, with soil moisture, vegetation type, shrub species characteristics, and growth patterns all influencing the direction and magnitude of shrub encroachment effects on soil nutrient reserves [15]. Therefore, the comprehensive consideration of climatic factors and soil conditions is crucial when studying shrub encroachment impacts on soil C and N reserves. However, research remains limited concerning the extent of shrub encroachment and its effects on C and N reserves across different grassland ecosystems.

The Altai Mountains are a unique environment with different plant and soil features. In recent decades, shrub encroachment has increased in this region, significantly altering the structure and function of local grassland ecosystems [16,17], and few studies have been conducted on the impact of shrub encroachment on soil carbon and nitrogen reserves in various grassland types in this region. In particular, the ecological effects of varying degrees of shrub encroachment across grassland types have not been systematically explored. Therefore, further research is needed to understand the impact of shrub encroachment on soil nutrient cycling in the Altai Mountains.

Thus, this study investigated five grassland types under varying extents of shrub encroachment in the Altai Mountains—mountain meadows, temperate meadow steppe, temperate steppe, temperate steppe desert, and temperate desert steppe—to explore its effects on soil organic carbon and nitrogen storage. Specifically, this study addresses the following: (1) how different extents of shrub encroachment affect soil properties and SOC and TN in these ecosystems; (2) whether these effects vary across grassland types; and (3) what key factors mediate the response of SOC_S_ and STN_S_ to shrub encroachment. This study’s findings provide a scientific basis for managing grassland shrub encroachment and ecological protection in the Altai Mountains.

## 2. Materials and Methods

### 2.1. Study Area

The Altai Mountains, which stretch about 1200 km from north to south, span across the borders of Russia, China, and Mongolia. Located in the heart of Eurasia (44°11′–46°20′ N, 84°31′–90°00′ E), the Altai Mountains in China lie along the middle section of the southern slope of the entire Altai Mountain range. The mountain range trends northwest to southeast and gradually decreases in elevation. It forms a crucial climate and natural boundary, acting as a barrier between the Pacific Ocean and Atlantic Ocean. The region experiences a continental temperate climate, with cold winters and thick snow. The average annual temperature ranges from −3.6 °C to 1.8 °C. Spring begins from late March to mid-April, and the region lacks a true summer season. The Altai Mountains display significant vertical differentiation in vegetation and biodiversity, hosting a diverse range of plant species across varying altitudes. At elevations between 736 and 1855 m (Table 1), *Spiraea salicifolia* L. is commonly found, along with other notable shrub species, such as *Spiraea hypericifolia* L., *Spiraea media* Schmidt, and *Spiraea media* Thunb. These shrubs are widely distributed across different grassland types, typically forming small, dense clusters. This variation in shrub species and distribution reflects the complex ecological conditions of the Altai Mountains, contributing to the region’s rich biodiversity. The soil types are brown calcic soil, black calcic soil, and chestnut calcic soil with silty and sandy texture [18].

### 2.2. Experiment Design

**Figure 1:**
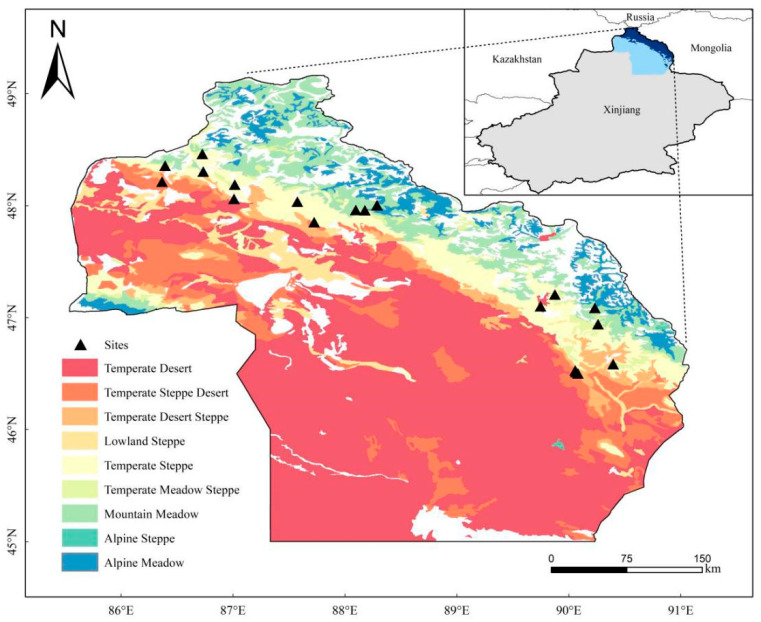
Locations of the study sites.

From July to August, 2023, 20 study sites were selected (Figure 1). Five grassland types were selected from the Altai Mountains: mountain meadows (MM), temperature meadow steppe (TMS), temperate steppe (TS), temperate steppe desert (TSD), and temperate desert steppe (TDS). According to the density, coverage, and shrub characteristics of *Spiraea* L. shrubs, three different shrub encroachment extents were selected and divided into low-, medium-, and high-shrub-encroachment grasslands. Non-shrub grassland was used as the control (CK), and homogeneous and flat natural grassland was selected as the test sample. Moreover, the principle of a complete randomized block design was adopted, and the low-, medium-, and high-shrub-encroachment grasslands were taken for soil sampling. This study used space-for-time substitution. According to the density, coverage, and shrub characteristics of the embroidery chrysanthemum shrub, a shrub coverage of less than 15% is low, a coverage of 15–30% is medium, and a coverage of more than 30% is high. The low, medium, and high shrub patch sizes were 0.11 m^2^, 0.18 m^2^, and 0.45 m^2^. The low, medium, and high shrub heights were 22 cm, 27 cm, and 45 cm. A natural grassland with uniform texture and a flat surface was selected as the experimental site for soil collection.

### 2.3. Soil Sampling and Analysis

Each treatment included 3 replicated plots (100 m × 100 m) separated by approximately 100 m. We measured the area of the shrub patches in a 5 m × 5 m plot at each 100 m × 100 m site. Five grassland quadrats (1 m × 1 m) were randomly placed at the center of each shrub plot. In each quadrat, all herbaceous plant individuals were recognized to the species level. The plant height was measured, and the coverage was calculated. All herb plants in the plot were picked at the soil surface to determine the aboveground biomass (AGB). Three replicates were chosen for each sampling site, using the 5-point sampling approach to reduce sampling heterogeneity, and then combined into a single sample. In total, 60 quadrats were sampled, with 45 quadrats for the encroached treatments and 15 quadrats for the non-encroached treatments. Soil was sampled between shrubs, at a distance of 0.5–1 m from the outer edge of the shrub. A 3.0 cm soil auger was used to gather soil samples from each quadrat at depths of 0–10 cm, 10–20 cm, and 20–40 cm. After removing coarse roots and debris, the soil was sieved using a 2 mm sieve and split into two portions based on soil analysis test indicators. The collected soil was subsequently taken to the laboratory, and the initial 200 g was analyzed to determine the soil properties. A second soil sample, about 100 g, was then taken to analyze the microbial biomass carbon (MBC) and microbial biomass nitrogen (MBN). During sampling, the soil was kept in a car refrigerator at 4 °C. Once returned to the laboratory, it was kept in the same refrigerator at 4 °C for immediate analysis.

A pH meter (PHS-3C) was used to determine the soil pH (soil-to-water ratio, 1:2.5). The soil bulk density (BD) was determined using the ring knife method (ring knife volume, 100 cm^3^). The soil water content (SWC) was measured using fresh soil samples with the oven drying method. The soil organic carbon (SOC) was assessed using a high-temperature combustion process and an enviro organic carbon analyzer (Vario EL III; Germany). The soil total nitrogen (TN) and total phosphorus (TP) were determined using a continuous flow autoanalyzer after digestion with concentrated sulfuric acid and perchloric acid (Bran Luebbe, AA3, Germany). The soil available nitrogen (AN) was determined using the alkali diffusion method, and the soil available phosphorus (AP) was determined using the NaHCO_3_ leaching–molybdenum antimony colorimetric method. The soil microbial biomass carbon (MBC) and microbial biomass nitrogen (MBN) were determined via chloroform fumigation–K_2_SO_4_ leaching, in which the carbon of extraction solution was determined using automatic total organic carbon. The amount of carbon in the extract was determined using an automatic total organic carbon analyzer, and the nitrogen was determined using a continuous flow analyzer.

### 2.4. Statistical Analyses

We used the species Shannon–Wiener diversity index (*H*) and Pielou evenness index (*E*) as our plant diversity indicators, which can be calculated as follows [19]:

Shannon–Wiener index:

(1) $$H=\sum_{i=1}^{s}Pi\ln Pi$$

Pielou index:

*E* = *H*/ln*S* (2)

where *S* represents the total number of plant species, and *Pi* is the importance value of the *i*th species, calculated according to the average values of the relative coverage, relative height, and relative biomass.

The SOC (stock of soil organic carbon) and STN (stock of soil total nitrogen) in k layers (0–10 cm, 10–20 cm, and 20–40 cm) were calculated, following the method outlined by Batjes [20]. The calculation formula is

(3) $$Ts=\sum_{i=1}^{k}C_{i}\times BD_{i}\times T_{i}$$

where *C_i_*, *BD_i_*, and *T_i_* represent the concentrations (of the SOC or STN), the bulk density, and the thickness of the *i*th layer, respectively.

To estimate the magnitude of the shrub encroachment effect on SOC_S_ and STN_S_, we used the relative interaction intensity (RII) index, as described by Armas et al. [21]. The RII was calculated using the following formula:

RII = (X_SE_ − X_CK_)/(X_SE_ + X_CK_) (4)

where X is the value of a given attribute (e.g., soil C or N), X_SE_ is the value under shrub encroachment, and X_CK_ is the value under control conditions. This index ranges from −1 to 1, with RII values > 0 indicating relatively greater values in the shrub encroachment than the shrub encroachment.

Statistical analyses were performed using IBM SPSS Statistics 25.0. One-way ANOVA and LSD multiple comparison tests were used to assess differences in soil nutrients, stoichiometry, and soil carbon and nitrogen stocks among different grassland types and varying degrees of shrub encroachment. Two-way ANOVA was also conducted to explore interaction effects. Differences were considered significant at *p* < 0.05. Pearson’s correlation analysis was conducted using Origin 2023 to identify linear relationships between variables. Structural equation models (SEMs) were constructed using Amos to analyze the pathways, through which biological and abiotic factors influence SOC_S_ and STN_S_. Map graphical representations were produced using ArcGIS 10.8 and Origin 2023 software. All values are presented as “mean ± standard error”.

## 3. Results

### 3.1. Shrub Encroachment Extent Effects on Soil Properties of Different Grassland Types

**Figure 2:**
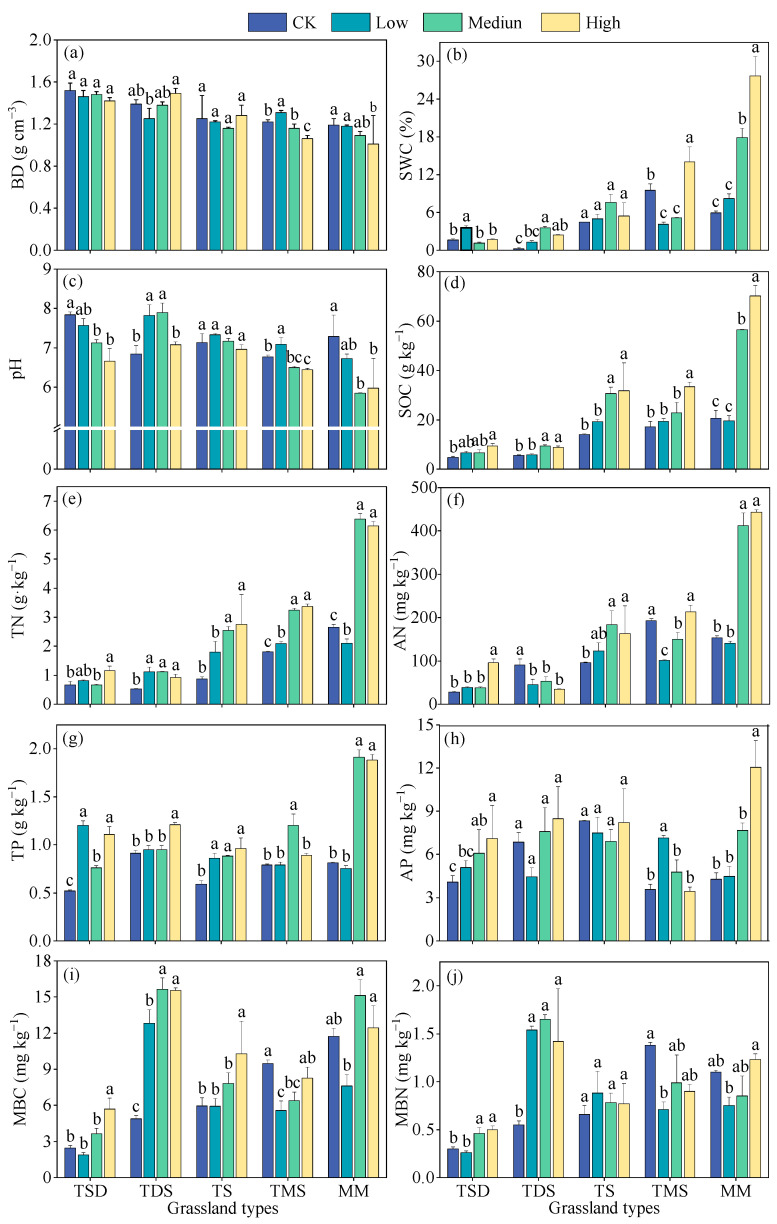
Effects of shrub encroachment extent on soil properties of different grassland types. (**a**) BD, soil bulk density (g cm^−3^); (**b**) SWC, soil water content (%); (**c**) pH; (**d**) SOC, soil organic carbon (g kg^−1^); (**e**) TN, soil total nitrogen (g kg^−1^); (**f**): AN, soil available nitrogen (mg kg^−1^); (**g**) TP, soil total phosphorus (g kg^−1^); (**h**) AP, soil available phosphorus (mg kg^−1^); (**i**) MBC, soil microbial carbon (mg kg^−1^); (**j**) MBN, soil microbial nitrogen (mg kg^−1^). Different letters indicate significant differences between the shrub encroachment extent (*p* < 0.05) based on Duncan’s multiple range test. Error bars indicate the SE of the mean. TDS: temperate desert steppe, TSD: temperate steppe desert, TS: temperate steppe, TMS: temperature meadow steppe and MM: mountain meadows.

The soil bulk density was not significantly affected by the shrub encroachment extent (*p* > 0.05), but the interaction among the grassland type, shrub encroachment extent, and grassland type was significant (*p* < 0.01) (Figure 2 and Table 2). Shrub encroachment significantly increased SOC, TN, TP, AN and AP (*p* < 0.05), while reducing soil moisture. Except for soil AP, which was not significant in the shrub encroachment extent (*p* < 0.05), SOC, TN, TP, AN and AP had significant interactions with grassland types in the shrub encroachment extent. The soil MBN showed an increasing trend with the shrub encroachment extent, but MBC showed significant differences in grassland types and shrub encroachment extent compared to grassland types, while MBN only showed significant differences in shrub encroachment extent compared to grassland types (*p* < 0.05).

### 3.2. Shrub Encroachment Extent Effects on Soil Stoichiometry of Different Grassland Types

**Figure 3:**
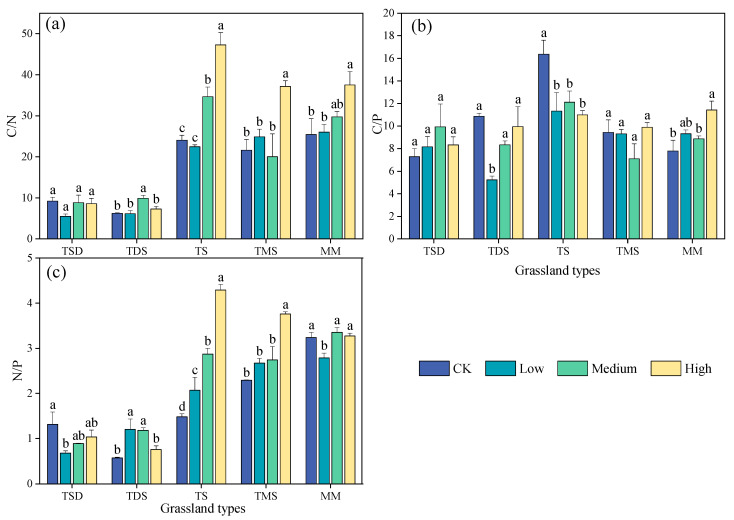
Shrub encroachment extent effects on soil stoichiometry of different grassland types. (**a**) C/N, (**b**) C/P, (**c**) N/P. Different letters indicate significant differences between the soil stoichiometry (*p* < 0.05) based on Duncan’s multiple range test. Error bars indicate the SE of the mean. TDS: temperate desert steppe, TSD: temperate steppe desert, TS: temperate steppe, TMS: temperature meadow steppe and MM: mountain meadows.

The soil C/N, C/P, and N/P were significantly different among the different grassland types, shrub encroachment extent, and interactions between grassland types and the shrub encroachment extent (*p* < 0.001) (Table 3). Overall, the soil C/N ratios showed a significant decreasing trend with the increasing shrub encroachment. Compared to the CK, the soil C/N significantly decreased by 16%, 10%, and 2% under low, medium, and high shrub encroachment, respectively. In contrast, the ratios of soil C/P and N/P increased with the extent of shrub encroachment. Specifically, soil C/P decreased by 2% under low shrub encroachment but increased significantly by 19% and 59% under medium and high shrub encroachment, respectively. Similarly, the soil N/P increased by 6%, 24%, and 47% with low, medium, and high shrub encroachment, respectively (Figure 3 and Table 3).

The soil stoichiometry characteristics varied among different grassland types. In general, there was no significant difference in soil C/N between TDS and TS (*p* > 0.05), but significant differences were observed in other grassland types, such as TDS, TMS, and MM (*p* < 0.05). In TDS, the soil C/N increased with shrub encroachment but remained lower than in CK. For the soil C/P, the highest value was observed in the CK soil in TDS (9.19), with the ratio first increasing and then decreasing with the degree of shrub encroachment. In TDS, the highest soil C/P ratio was observed under high shrub encroachment. Similarly, the soil C/P increased with shrub encroachment in TS, TMS, and MM (*p* < 0.05), reaching across grassland types. In TSD, TMS, and TDS, the soil N/P increased with the extent of shrub encroachment (*p* < 0.05), but in TDS, the N/P ratio in non-encroached soil was higher than in encroached soil. In TDS, the soil N/P decreased with the extent of shrub encroachment (*p* < 0.05), consistently remaining higher than in the CK soil. There was no significant difference in the soil N/P in MM (*p* > 0.05), indicating that shrub encroachment did not substantially affect the N/P ratio in this grassland type.

### 3.3. Shrub Encroachment Extent Changes in SOC_S_ and STN_S_

**Figure 4:**
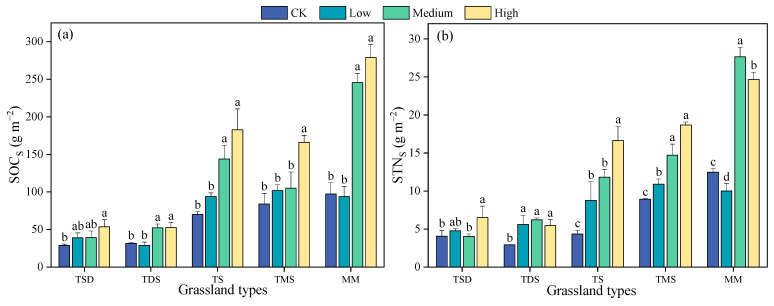
SOC_S_ (**a**) and STN_S_ (**b**) at the 0–40 cm depth at the shrub encroachment extent in five grassland types. Different letters indicate significant differences between the shrub encroachment extent (*p* < 0.05) based on Duncan’s multiple range test. Error bars indicate the SE of the mean. TDS: temperate desert steppe, TSD: temperate steppe desert, TS: temperate steppe, TMS: temperature meadow steppe and MM: mountain meadows.

There were significant differences in SOC_S_ and STN_S_ among different grassland types, shrub encroachment extents, and the interaction between grassland types and shrub encroachment extent (*p* < 0.001) (Table 3). Both SOC_S_ and STN_S_ increased significantly with the extent of shrub encroachment (Figure 4 and Table 3). Overall, compared to CK, SOC_S_ increased by 16%, 77%, and 129% under low, medium, and high shrub encroachment, respectively, while STN_S_ increased by 43%, 94%, and 127%, respectively.

Specifically, in TDS, SOC increased by 34%, 36%, and 86% under low, medium, and high shrub encroachment, respectively. However, in TDS, a slight decrease of 8% was observed in SOC_S_ under low shrub encroachment, while SOC_S_ increased by 66% and 67% under medium and high encroachment, respectively. In TS, SOC_S_ increased by 34%, 105%, and 161% under low, medium, and high shrub encroachment, respectively. In TMS, SOC increased by 21%, 25%, and 98% in low, medium, and high encroachment, respectively. In contrast, MM showed a 4% decrease in low SOC_S_, followed by a significant increase of 153% in medium SOC_S_ and of 187% in high SOC_S_.

For STN_S_, in TSD, medium STN_S_ decreased by 1%, while low and high STN increased by 17% and 60%, respectively. In TDS, STN increased by 93%, 141%, and 88% under low, medium, and high shrub encroachment, respectively. Similarly, in TS, STN_S_ increased by 101%, 172%, and 283% under low, medium, and high shrub encroachment, respectively. In TMS, STN_S_ increased by 22%, 64%, and 109% under low, medium, and high shrub encroachment, respectively. In MM, low STN decreased by 20%, while medium and high STN_S_ increased by 123% and 98%, respectively. These results highlight the significant effects of shrub encroachment on SOC_S_ and STN_S_, with variation across different grassland types and encroachment intensities.

**Figure 5:**
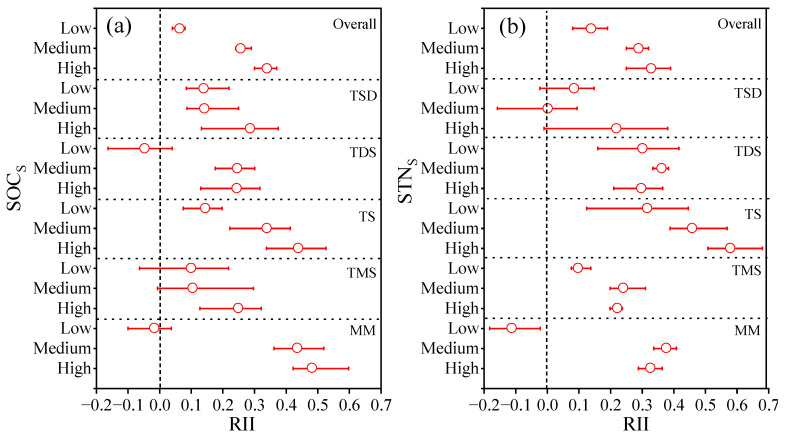
Relative interaction intensity (RII) for SOC_S_ (**a**) and STN_S_ (**b**) under shrub encroachment for five grassland types. TDS: temperate desert steppe, TSD: temperate steppe desert, TS: temperate steppe, TMS: temperature meadow steppe and MM: mountain meadows.

Overall, the SOC_S_ and STN_S_ in different grassland types increased with the shrub encroachment extent, and the RII values were highest with high shrub encroachment (Figure 5). The SOC_S_ RII values were 0.06, 0.25, and 0.34 under low, medium, and high shrub encroachment, respectively, and the STN_S_ RII values were 0.14, 0.29, and 0.33 under low, medium and high shrub encroachment, respectively. The SOC_S_ showed a negative effect under low shrub encroachment in TDS and MM but a positive effect in other grassland types. The STN_S_ showed a negative effect under low and medium shrub encroachment in TSD and MM, respectively but a positive effect in other grassland types.

### 3.4. Relationship Between SOC_S_, STN_S_ and Biological and Abiotic Factors

**Figure 6:**
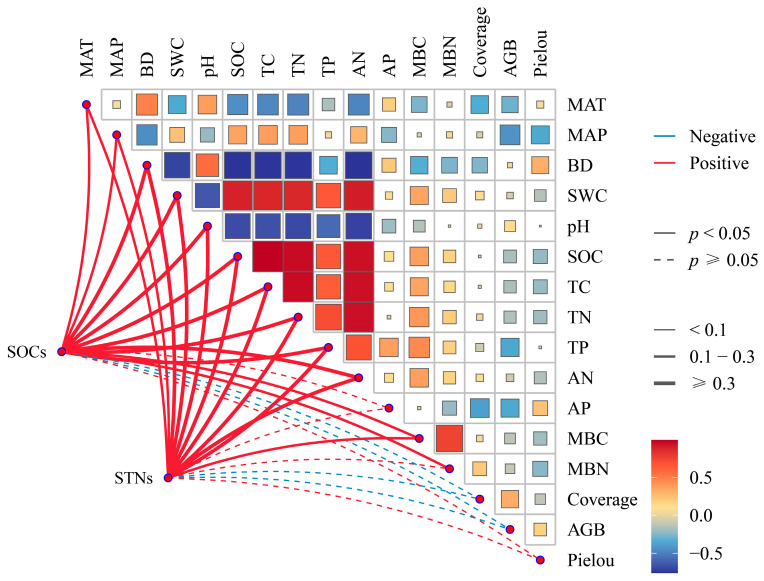
Correlation matrix of biological and abiotic factors as well as SOC_S_ and STN_S_ in different grassland types. MAT, mean annual temperature; MAP, mean annual precipitation; BD, bulk density (g cm^−3^); SWC, soil water content (%); SOC, soil organic carbon (g kg^−1^); TC, soil total carbon (g kg^−1^); TN, soil total nitrogen (g kg^−1^); TP, soil total phosphorus (g kg^−1^); AN, soil available nitrogen (mg kg^−1^); AP, soil available phosphorus (mg kg^−1^); MBC, soil microbial biomass carbon (mg kg^−1^); MBN, soil microbial biomass nitrogen (mg kg^−1^); Coverage, herbaceous vegetation cover (%); AGB, aboveground plant biomass (g m^−2^); Pielou, Pielou’s index.

The correlation matrix revealed substantial relationships between SOC_S_, STNS, and various other soil parameters (Figure 6). SOC_S_ is positively correlated with MAT, MAP, BD, SWC, pH, SOC, TC, TN, TP, AN, and MBC (*p* < 0.01), but negatively correlated with coverage, AGB, and Pielou’s index. A similar pattern was shown for STN_S_, which has positive correlations with MAT, MAP, BD, SWC, pH, SOC, TC, TN, TP, AN, and MBN but negative correlations with coverage, AGB, and Pielou’s index (*p* > 0.05).

**Figure 7:**
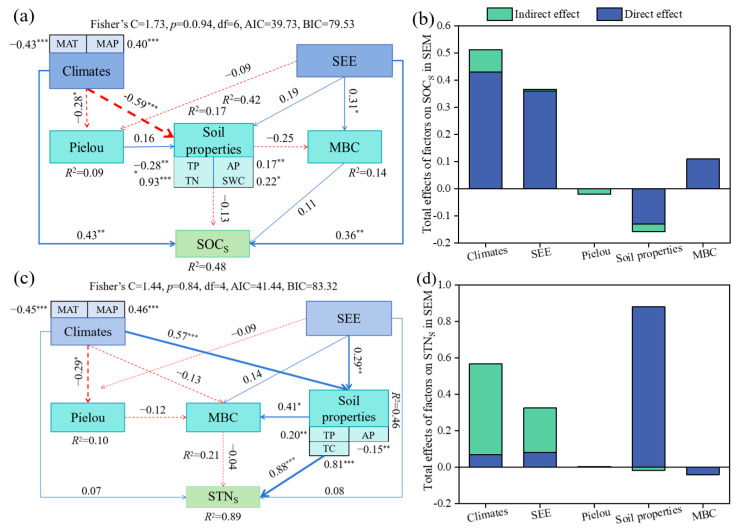
Effects of shrub encroachment on SOC_S_ and STN_S_. (**a**,**c**), calculated using the structural equation model of path analysis, according to the standardized path coefficients. (**b**,**d**), direct and indirect effects of all factors. The numbers adjacent to arrows, path coefficients, which are analogous to partial correlation coefficients, are indicative the effect size of the relationship and may be positive (blue) or negative (red) The significant levels are the following: *, *p* < 0.05; **, *p* < 0.01; ***, *p* < 0.001. SEE, shrub encroachment extent; MAT, mean annual temperature; MAP, mean annual precipitation; TC, soil total carbon (g kg^−1^); TP, soil total phosphorus (g kg^−1^); AP, soil available phosphorus (mg kg^−1^); MBC, soil microbial biomass carbon (mg kg^−1^).

Furthermore, structural equation modeling (SEM) was used to explore the pathways through which climate, SEE, soil properties, and MBC influence SOC_S_ and STN_S_ (Figure 7). The results show that climate and SEE, or indirect changes in plant diversity and soil physical and chemical properties, affected SOC_S_ and STN_S_. The SEE, soil properties, plant diversity, and MBC explain 48% of the variation in SOC_S_ and 89% of the variation in STN_S_, respectively. Important factors affecting SOC_S_ and STN_S_ were assessed by calculating the direct and indirect standardized effects of the five explanatory variables on SOC_S_ and STN_S_ (Figure 7b,d). The results show that climate and SEE were directly the largest factors influencing changes in SOC_S_, followed by soil properties and MBC, while plant diversity had the smallest effect. The indirect effect of climate was the largest factor influencing SOC_S_ changes, followed by soil properties and MBC, while plant diversity affected it the least. The direct effect of soil properties was the largest factor influencing the change in STN_S_, followed by climate and SEE, while plant diversity affected it the least. The indirect effect of climate and SEE was the largest factor influencing the change in STN_S_ followed by soil properties.

## 4. Discussion

### 4.1. Effects of Shrub Encroachment on SOC_S_ and STN_S_

Shrub encroachment has been recognized as a major ecological process with profound effects on grassland ecosystems, particularly regarding SOC_S_ and STN_S_ [22]. Our results confirm that shrub encroachment significantly enhances both SOC_S_ and STN_S_ across various grassland types in the Altai Mountains. This is consistent with previous studies, especially those indicating that shrub encroachment can increase soil carbon and nitrogen reserves through several mechanisms, such as changes in plant composition, litter input, and microbial activity [23,24]. The SEM results demonstrate that SEE and climatic factors (MAT and MAP) have direct and indirect effects on SOC_S_ and STN_S_. Soil properties, particularly TN, TP, and SWC, were the strongest mediators of SOC_S_ and STN_S_ accumulation. Additionally, MBC played a critical role in mediating the indirect effects of SEE and climate; in contrast, plant diversity (Pielou’s index) had a relatively minor influence. High levels of encroachment resulted in the most significant increases in SOC_S_ (up to 129%) and STN_S_ (up to 127%), suggesting that as shrub density increases, the ability of the soil to store organic carbon and nitrogen also increases. This is in line with the results of Zhao et al. [10] who revealed that shrub encroachment enhance SOC_S_ and STN_S_ due to direct and indirect influence. These effects were most pronounced in grassland types such as MM and TS, which showed substantial increases in both SOC_S_ and STN_S_ as shrub encroachment progressed. In contrast, some grassland types, particularly TDS, showed less pronounced or even negative effects at lower levels of encroachment, likely due to differences in environmental conditions (e.g., soil texture, moisture availability, and plant species composition) and shrub species characteristics. The SEM analysis also highlighted nitrogen availability as a critical driver of SOC_S_ and STN_S_ accumulation. Shrub species, many of which are nitrogen-fixing plants, play a pivotal role in enhancing STN availability, which promotes nutrient cycling and organic matter accumulation. The model revealed that shrub encroachment extent and grassland type had direct effects on soil available nitrogen, which in turn influenced STN_S_. This pathway underscores the significance of shrub encroachment in enhancing both SOC_S_ and STN_S_ in encroached grasslands.

Shrub encroachment not only affects the physical structure of the soil but also alters its chemical properties, particularly through changes in soil pH [25], microbial activity [26], and nutrient cycling processes [24]. The observed positive correlations between soil water content, SOC, TN, and MBC further emphasize the role of soil moisture and microbial activity in driving these changes. In contrast, negative correlations between pH and BD with SOC and STN suggest that higher pH and greater BD may suppress microbial activity and nutrient cycling, thereby limiting the accumulation of organic carbon and nitrogen in the soil [27]. These findings suggest that shrub encroachment, in modifying soil properties and microbial activity, can enhance soil fertility and carbon sequestration potential in grassland ecosystems, particularly in more arid or nutrient-poor regions.

### 4.2. Soil Stoichiometry and Its Response to Shrub Encroachment

In addition to the changes in SOC_S_ and STN_S_, shrub encroachment significantly altered soil stoichiometry [28]. The ratios of C/N, C/P, and N/P provide valuable insights into nutrient cycling and ecosystem functioning [29]. Our results show a significant decrease in the C/N ratio with increasing shrub encroachment, indicating that the accumulation of nitrogen in soil outpaces carbon accumulation, which is a crucial shift for nutrient cycling in grassland ecosystems. The decrease in the C/N ratio is often associated with increased nitrogen availability due to enhanced microbial activity and decomposition processes in shrub-dominated soils [8,30]. The increase in both the C/P and N/P ratios with shrub encroachment, especially at higher levels of encroachment, reflects a shift toward greater phosphorus and nitrogen availability relative to carbon. This shift in stoichiometry ratios is indicative of the increased input of litter and organic matter from shrub growth, which contains higher levels of nutrients compared to herbaceous vegetation [13]. These findings suggest that shrub encroachment not only enriches soil with carbon and nitrogen but also alters the nutrient balance, potentially influencing plant species composition and ecosystem productivity over time. Interestingly, the patterns of soil stoichiometry change were not uniform across all grassland types. For instance, while the C/P ratio increased in most grassland types, it exhibited more complex trends in some, such as the TDS, where the effect of shrub encroachment on phosphorus availability appeared more pronounced in areas with medium and high encroachment. This variability may be related to the specific shrub species present, soil type, and moisture conditions, which all play roles in determining nutrient dynamics [31]. Additionally, soil stoichiometry changes were closely linked to shifts in microbial communities and their nutrient utilization strategies, which warrants further investigation into the microbial drivers behind these changes [32].

The stoichiometric changes in nutrients become more prominent at higher shrub encroachment levels, since shrub litter provides more nitrogen and phosphorus than herbaceous vegetation. The nutrient availability might increase as a result of this shift, thus affecting ecosystem productivity and plant species distribution. Shrub encroachment affects soil stoichiometry differentially, depending on the specific shrub kinds and native soil types, as well as local moisture levels in distinct grassland habitats [33]. Research has shown that shrub encroachment systems feature complex processes involving ecosystem nutrients, while microbial communities serve as essential mediators of these reactions.

### 4.3. Implications for Ecosystem Management and Climate Change Mitigation

The results of this study have significant implications for grassland management and ecosystem restoration, particularly in the context of climate change and desertification. The positive effects of shrub encroachment on SOC_S_ and STN_S_ suggest that shrub encroachment may serve as important carbon and nitrogen sinks, potentially mitigating climate change by sequestering large amounts of greenhouse gases. Given that shrub encroachment is often associated with degraded or overgrazed grasslands, understanding how shrub encroachment ecosystems contribute to carbon and nitrogen storage is crucial for land restoration efforts [7,9]. However, it is important to recognize that the ecological benefits of shrub encroachment may vary depending on the degree of encroachment, grassland type, and local environmental conditions. In some cases, excessive shrub encroachment could lead to negative outcomes, such as reduced grassland productivity, loss of biodiversity, and altered hydrological cycles [34]. Therefore, careful management is required to balance the benefits of shrub encroachment with the need to maintain the ecological integrity of grassland ecosystems.

Our research supports adaptive management plans that should account for the extent of shrub encroachment together with site-specific habitat characteristics. Managed shrub growth expansion should receive support when it improves soil quality and carbon accumulation, but shrub containment strategies are necessary when shrub presence poses threats to biodiversity or water resources. The implementation of customized management approaches will optimize ecological advantages alongside the reduction in potential negative outcomes.

Given the transformative potential of shrub encroachment, further research is essential to explore the long-term sustainability of SOC and STN enhancement, the role of different shrub species in modulating soil nutrient dynamics, and the complex interactions between shrubs, soil microbes, and plant communities under changing climatic conditions. Such studies will provide deeper insights into the mechanisms of nutrient cycling and the resilience of grassland ecosystems to environmental changes.

## 5. Conclusions

This study emphasizes the importance of shrub encroachment in enhanced soil carbon sequestration and modifying nitrogen cycling across several grassland types in the Altai Mountains. Our data reveal that shrub encroachment causes significant increases in SOC_S_ and STN_S_, as well as changes in soil stoichiometry, namely in the C/N, C/P, and N/P ratios. These results show that shrub encroachment increases SOC_S_ and STN_S_ while modifying the nutrient balance in grasslands, making them more suitable for long-term carbon sequestration. In regions with medium to high shrub encroachment, the stabilization of the C/N ratio over time suggests that nutrient accumulation levels off as the ecosystem matures. These findings provide valuable insights into the complex interactions between shrub encroachment, soil nutrients, and ecosystem functioning. Additionally, shrub encroachment appears to enhance plant resilience to environmental stresses, particularly drought, by improving soil nutrient dynamics and water retention. In summary, shrub encroachment is a key driver of soil carbon and nitrogen dynamics in grasslands, promoting carbon sequestration and nutrient cycling, particularly in arid and semi-arid regions. As global climate change continues to reshape grassland ecosystems, understanding the long-term impacts of shrub encroachment will be critical for managing changes in biogeochemical cycles, ecosystem services, and ecosystem health.

## Figures and Tables

**Table 1:** Background information of soil sampling sites. SEE: shrub encroachment extent. TDS: temperate desert steppe, TSD: temperate steppe desert, TS: temperate steppe, TMS: temperature meadow steppe and MM: mountain meadows.

	SEE	Longitude (°)	Latitude (°)	Altitude (m)	MAT (°C)	MAP (mm)	Clay (%)	Silt (%)	Sand (%)	Dominant Plants
TSD	CK	90.0623	46.5148	830	4.4	166.4	5.71	62.73	31.56	*Seriphidium gracilescens*
	Low	87.7235	47.8543	832	4.7	205.7	3.88	24.59	71.53	*Festuca ovina*
	Medium	86.3645	48.2155	736	5.3	304.0	9.83	40.91	49.26	*Seriphidium gracilescens*
	High	90.0548	46.5278	832	4.4	166.4	6.75	62.41	30.84	*Seriphidium gracilescens*
TDS	CK	89.7463	47.1020	1212	1.3	166.6	7.07	67.88	25.05	*Seriphidium gracilescens*
	Low	87.0078	48.0623	853	5.0	249.7	2.20	26.80	71.00	*Seriphidium gracilescens*
	Medium	90.0798	46.5024	1243	4.3	166.4	9.95	50.94	39.11	*Festuca ovina*
	High	90.3947	46.5851	1259	2.3	164.8	2.76	17.71	79.53	*Artemisia frigida*
TS	CK	89.8744	47.2067	1221	1.7	152.3	7.38	70.26	22.36	*Festuca ovina*
	Low	87.5740	48.0366	1262	0.9	232.8	5.97	36.77	57.25	*Carex tristachya*
	Medium	86.7255	48.4629	1110	1.4	312.6	9.83	59.05	31.12	*Carex tristachya*
	High	86.7337	48.3056	1266	1.9	318.8	9.95	59.93	30.12	*Carex tristachya*
TMS	CK	88.0958	47.9620	1089	3.1	209.8	6.96	75.39	17.66	*Carex tristachya*
	Low	90.2601	46.9417	1533	0.5	162.2	7.91	45.35	46.74	*Festuca ovina*
	Medium	87.0148	48.1884	1191	2.7	271.1	1.52	28.75	69.74	*Carex tristachya*
	High	86.3922	48.3581	1251	3.0	354.1	13.96	72.10	13.94	*Carex tristachya*
MM	CK	88.2843	48.0049	1698	−2.3	237.6	5.11	74.05	19.52	*Carex tristachya*
	Low	90.2320	47.0876	1855	−1.8	174.3	7.61	43.94	48.46	*Carex tristachya*
	Medium	88.2873	48.0040	1732	−2.3	237.6	7.73	78.97	13.31	*Carex tristachya*
	High	88.1773	47.9607	1589	2.2	210.4	7.49	75.15	17.36	*Carex tristachya*

**Table 2:** Effects of grassland types (GT), shrub encroachment extent (SEE), and GT×SEE on soil properties in different treatments. Results of the ANOVA analyses (F-values) for the effects of GT, SEE, and their interaction (GT × SEE) on soil properties under different treatments. *, *p* < 0.05; **, *p* < 0.01; ***, *p* < 0.001. BD, SWC, SOC, TN, AN, TP, AP, MBC and MBN are the same as above.

	BD	SWC	pH	SOC	TN	TP	AN	AP	MBC	MBN
GT	39.41 ***	122.89 ***	15.58 ***	232.71 ***	495.96 ***	142.61 ***	229.32 ***	11.65 ***	24.54 ***	2.59
SEE	1.97	8.89 ***	4.44 **	17.93 ***	20.58 ***	19.33 ***	7.13 ***	4.20 *	1.178	0.14
GT × SEE	6.50 ***	6.60 ***	3.17 **	9.33 ***	13.45 ***	10.44 ***	6.78 ***	4.36 ***	21.91 ***	6.09 ***

**Table 3:** Effects of GT, SEE, and GT × SEE on soil stoichiometry, SOC_S_, and STN_S_ under different treatments. Results of the ANOVA analyses (F-values) for the effects of GT, SEE, and their interaction (GT × SEE) on soil stoichiometry under different treatments. *, *p* < 0.05; ***, *p* < 0.001.

Factor	C/N	C/P	N/P	SOC_S_	STN_S_
GT	12.64 ***	116.85 ***	222.45 ***	100.12 ***	87.83 ***
SEE	3.05 *	24.42 ***	34.50 ***	59.13 ***	55.02 ***
GT × SEE	3.65 ***	5.48 ***	16.49 ***	12.14 ***	10.06 ***

## Data Availability

Data will be made available on request.
